# Sodium Butyrate Alleviates Intestinal Inflammation in Mice with Necrotizing Enterocolitis

**DOI:** 10.1155/2021/6259381

**Published:** 2021-10-12

**Authors:** Qian Sun, Yan-Chun Ji, Zheng-Li Wang, Xiang She, Yu He, Qing Ai, Lu-Quan Li

**Affiliations:** ^1^Department of Neonatology, Children's Hospital of Chongqing Medical University, Chongqing, China; ^2^National Clinical Research Center for Child Health and Disorders, Chongqing, China; ^3^Key Laboratory of Children's Development and Disorders, Ministry of Education, Chongqing, China; ^4^National International Science and Technology Cooperation Base for Development and Critical Disorders in Children, Chongqing, China; ^5^Key Laboratory of Pediatrics in Chongqing, Chongqing 400014, China

## Abstract

**Objective:**

To determine the role of sodium butyrate in intestinal inflammation via regulation of high-mobility group box-1 (HMGB1), we analyzed the potential mechanism in necrotizing enterocolitis (NEC) in a neonatal mouse model.

**Methods:**

A NEC model was created with hypoxia and cold exposure and artificial overfeeding. C57BL/6 neonatal mice were randomized into three groups: the control, untreated NEC, and sodium butyrate (150 mM)-pretreated NEC groups. Pathological variations in ileocecal intestinal tissue were observed by HE staining and scored in a double-blind manner. The mRNA expression levels of HMGB1, Toll-like receptor 4 (TLR4), nuclear factor-*κ*B (NF-*κ*B), and inflammatory cytokines in intestinal tissues were determined by quantitative real-time PCR. The protein levels of HMGB1 and associated cytokines in intestinal tissues were evaluated using ELISA. The relative protein expression levels of TLR4 and NF-*κ*B in intestinal tissues were quantified by western blot.

**Results:**

Sodium butyrate administration improved the body weight and survival rate of NEC mice; relieved intestinal pathological injury; reduced the intestinal expression of HMGB1, TLR4, NF-*κ*B, interleukin- (IL-) 1*β*, IL-6, IL-8, and TNF-*α*; and increased the intestinal expression of IL-10 (*P* < 0.05). Treatment with butyrate decreased the proportion of opportunistic *Clostridium_sensu_stricto_1* and *Enterococcus* and increased the proportion of beneficial *Firmicutes* and *Lactobacillus* in the NEC model.

**Conclusions:**

Sodium butyrate intervention relieves intestinal inflammation and partially corrects the disrupted intestinal flora in mice with NEC.

## 1. Introduction

Necrotizing enterocolitis (NEC) is one of the most common and serious diseases of the digestive system of newborns. This disease mainly occurs in premature and low-birth-weight infants, with an occurrence rate of approximately 9–13%, seriously affecting the survival and long-term prognosis of children [1]. In the past 50 years, the overall survival rate has not changed significantly, with an average mortality rate of 20–30% for infants with NEC and 50% for those requiring surgical treatment [2]. Reducing the occurrence of NEC and applying positive and effective prevention and treatment measures are still major challenges worldwide.

NEC is caused by multiple factors, and an excessive intestinal inflammatory response and altered microbiota are both important in the pathogenesis of this disease [3]. In NEC rat models, proinflammatory cytokine production is strongly linked with enteric inflammation and NEC [4]. In NEC infants, the intestinal flora diversity is often lower than that in unaffected infants and is mainly characterized by an increase in the relative abundance of *Proteobacteria* and a decrease in the relative abundance of *Firmicutes* and *Bacteroides* [5].

Butyric acid, an aliphatic carboxylic acid with a four-carbon tail, can be produced by intestinal *Firmicutes*-mediated decomposition of oligosaccharides, primarily in the upper part of the colon near the ileal junction (the most common site of NEC) [6, 7]. Past studies have revealed that sodium butyrate has several beneficial functions, such as anticancer activity and the ability to improve intestinal function [8]. Research suggests that exogenous butyrate can downregulate inflammation and protect the intestine from NEC [9]. However, the effect of butyrate administration on bacterial flora in NEC has rarely been studied.

Based on this information, we hypothesized that sodium butyrate treatment would alleviate intestinal inflammation and flora dysbiosis in mice with NEC. To test this hypothesis, we established a NEC animal model and treated these animals with sodium butyrate to investigate the moderating effect of sodium butyrate on intestinal inflammation, related cytokines, and intestinal flora. The generated evidence will lay the foundation for further research to elucidate the detailed mechanism.

## 2. Materials and Methods

### 2.1. Neonatal NEC Model and Experimental Design

All operations performed on the animals were approved by the Animal Ethics Committee at Chongqing Medical University. The neonatal NEC model was established as reported by Gopalakrishna et al., with some adjustments, and the following method description partially reproduces their phrasing [10, 11]. Newborn 3-day-old male or female C57BL/6 mice weighing 1.5–3.0 g were randomized into three groups: the control, untreated NEC, and sodium butyrate-treated NEC groups. Sodium butyrate (Shanghai Biological Engineering, China) was formulated as a 150 mM solution at pH 7.62 (weakly alkaline). Liu et al. reported that the administration of 150 mM butyrate by enema can protect the intestine from NEC in vivo [9]; therefore, we chose this dose of sodium butyrate for our experiments, but further work is needed to investigate the most effective administration method and concentration of butyrate. From the 3rd day after birth, mice in the experimental groups received daily intragastric PBS or 150 mM butyrate solution at 0.03 ml/g for 7 days. Then, the neonatal NEC model was established for 3 days. The control group was fed freely by their mothers. Mixed formula milk (Similac Advance (Abbott Nutrition, USA)/Esbilac puppy milk replacer (PetAg, USA) = 1.7) was given to both NEC groups by gavage 5 times per day at 4 h intervals and skipped one feeding at 4:00 am per day on 3 consecutive days at amounts based on body weight. The mice were placed in a homemade hypoxia box filled with 100% N_2_ for 90 s and then placed in a refrigerator at 4°C for 10 min. Hypoxia and cold exposure occurred three times per day (at 10:00, 14:00, and 22:00), while the control group was not exposed to either stimulus. The NEC model was considered established 3 days after the last feeding. All mice were fasted and not disturbed for 12 h after the last feeding, and intestinal tissues and contents were subsequently collected as test samples.

### 2.2. Histological Examination

Three groups of pups were decapitated on the fourth day after NEC induction. For each mouse, the intestine from the lower end of the duodenum to the ileocecum was removed, and NEC-like lesions such as gas accumulation, hemorrhage, and necrosis were observed with the naked eye. Fresh proximal ileocecum (1–2 cm) samples were immediately fixed in 4% paraformaldehyde. Then, the sections were dehydrated, embedded in paraffin, sectioned into 4 *μ*m tissue slices, stained with hematoxylin and eosin (HE), and sealed. The histopathological changes in the intestine were observed under an optical microscope. NEC was scored based on changes in intestinal tissue using the double-blind observational method reported by Yu et al.: 0: no damage; 1: epithelial cell lifting or separation; 2: necrosis to the midvillous level; 3: necrosis of the entire villus; 4: transmural necrosis. Animals with a histologic tissue injury score ≥ 2 were considered positive for NEC [12].

### 2.3. Quantitative Polymerase Chain Reaction (QPCR)

RNA was obtained from intestinal tissue using RNAiso Plus (Takara, Japan) and converted by reverse transcription (RT) to cDNA using a PrimeScript RT reagent kit with gDNA Eraser (Takara, Japan). QPCR was performed using a TB Green Premix Ex Taq II Kit (Takara, Japan) and BIO-RAD CFX96 Real-Time PCR detector. The specific primer sequences used are listed in Table 1. The housekeeping gene GAPDH was used to normalize mRNA expression, and relative expression was calculated as the mean 2^−*ΔΔ*Ct^.

### 2.4. Enzyme-Linked Immunosorbent Assay (ELISA)

According to the instructions of NP-40 lysis buffer (Beyotime, China), the corresponding volume of protease inhibitor PMSF (Beyotime, China) was added into NP-40 lysis buffer to ensure that the final concentration of PMSF was 1 mM. The frozen intestinal tissue was taken out from the refrigerator at -80°C. 100 *μ*l NP-40 lysis buffer containing PMSF was added per 10 mg intestinal tissue and homogenized with ice electric homogenizer for 10 min; then, the tissue homogenate was centrifuged at 4°C (14000g, 10 min). The supernatant was taken and stored at -80°C. The concentrations of high-mobility group box-1 (HMGB1), interleukin- (IL-) 10, tumor necrosis factor-*α* (TNF-*α*), IL-1*β*, IL-6, and IL-8 in the supernatant of mouse intestinal tissue were measured with mouse HMGB1 (Arigobio, China) and IL-10, TNF-*α*, IL-1*β*, IL-6, and IL-8 ELISA kits (4A BIOTECH, China) and a BioTek Synergy H1 instrument (USA) following the manufacturers' directions.

### 2.5. Western Blot Assay

Distal ileum tissues were homogenized for 20 min with an electrically powered instrument in a solution containing PMSF and NP-40 (Beyotime, China). Then, an enhanced BCA protein assay kit (Beyotime, China) was used for protein quantitation. The samples were mixed with loading buffer and boiled for 5 min to denature the proteins, which were then separated by SDS-PAGE and transferred onto PVDF membranes. After blocking nonspecific binding at room temperature in fast blocking buffer for 10 min, the membranes were incubated overnight at 4°C with specific antibodies against *β*-actin (1 : 10,000), Toll-like receptor 4 (TLR4, 1 : 1000), and nuclear factor-*κ*B (NF-*κ*B, 1 : 7500) (ZENBIO Biotechnology, China). After the membrane was washed thoroughly with TBST, it was incubated for 2 h with a horseradish peroxidase- (HRP-) conjugated secondary antibody. The membrane was thoroughly washed with TBST, and then, the bands were detected using enhanced hypersensitive electrochemiluminescence (ECL) western blotting detection reagents (ZENBIO Biotechnology, China) on a BIO-RAD ChemiDoc™ Touch Imaging System. Relative quantification of the levels of target proteins compared to those of *β*-actin was accomplished with ImageJ software.

### 2.6. Immunohistochemistry

Paraffin-embedded intestinal samples were deparaffinized in xylene and dehydrated in a series of ethanol solutions. For antigen retrieval, tissue slices were covered with citric acid antigen retrieval buffer (pH 6.0) and heated in a microwave oven for 25 min. Endogenous peroxidases were blocked, and nonspecific binding was blocked with 3% BSA for 30 min. Then, sections were incubated with primary anti-TLR4 and anti-NF-*κ*B antibodies overnight at 4°C, rinsed in PBS, and subsequently incubated at room temperature for 1 h with the appropriate HRP-labeled secondary antibody. After DAB coloration, hematoxylin was added to stain the nucleus, the sections were dehydrated, and cover slips were added. Random fields were qualitatively assessed using a microscope (200x magnification). The hematoxylin-stained nucleus appeared blue, and the DAB (brown-yellow)-stained area was considered positive for antigen.

### 2.7. Fecal Sample Collection and Microbiota Analysis

The ileum and colon contents of mice were lavaged with 1000 *μ*l PBS and collected in 1.5 ml sterile tubes. Then, vortex oscillating intestinal contents for 5 min and centrifuging at 4°C (14000g, 10 min), the sediment was collected and used for fecal sample microbiota analysis. Using a QIAamp Fast DNA Stool Kit (Qiagen, Germany), fecal microbiota genomic DNA was extracted following the manufacturer's directions. DNA was evaluated by 1% agarose gel electrophoresis, and the concentration and purity were determined to ensure quality. The V3-to-V4 region was amplified with the designated primers under the following protocol: 3 min at 95°C; 27 cycles of denaturation (30 s at 95°C), annealing (30 s), and extension (45 s at 72°C); additional extension (10 min at 72°C); and hold at 10°C. The product was isolated by 2% agarose gel electrophoresis, recovered using an AxyPrep DNA gel extraction kit (Axygen Biosciences), and quantified with a Quantus™ Fluorometer (Promega, USA). A database was established, and PCR products were sequenced on the Illumina MiSeq platform. The original data were processed. Briefly, reads containing bases with a quality score < 20 were truncated, and sequences longer than 10 bp were combined in an overlapping manner. In the overlapping region of the spliced sequences, reads exceeding the maximum mismatch ratio of 0.2 were deleted. Using UPARSE (version 7.1), the processed sequences were divided into operational taxonomic units (OTUs), and bioinformatics data were clustered based on OTUs at a 97% similarity threshold.

### 2.8. Statistical Analysis

GraphPad Prism (version 8.3.0) was applied to analyze all data and test for a normal distribution. Normally distributed data were presented as the mean ± SD, and significance was determined using one-way analysis of variance (ANOVA) or two-way ANOVA. The median and interquartile range (IQR) were used to describe nonnormally distributed data, and differences were determined by the Kruskal-Wallis test. *P* < 0.05 was considered to indicate statistical significance.

## 3. Results

### 3.1. Sodium Butyrate Reduced the Severity of NEC in a Mouse Model

#### 3.1.1. Changes in Body Weight and Survival Rate

**Figure 1:**
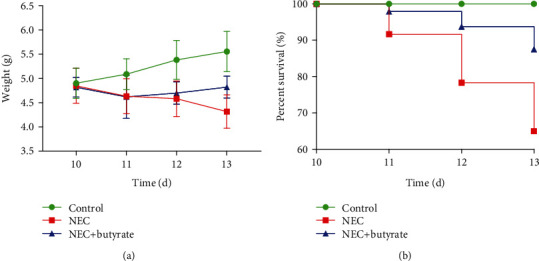
(a) Body weight changes in newborn mice in the three groups. Number of samples: control (*n* = 8), NEC (*n* = 8), and NEC+butyrate (*n* = 8). Statistical analysis: two-way ANOVA multiple comparisons method. (b) Survival curves of newborn mice in the three groups. The survival rate was estimated during modeling, and the results are shown as a Kaplan-Meier plot. Numbers of samples at the beginning: control (*n* = 40), NEC (*n* = 60), and NEC+butyrate (*n* = 48). Statistical analysis: log-rank (Mantel-Cox) test (*P* < 0.05). Control: normal control; NEC: necrotizing enterocolitis; NEC+butyrate: sodium butyrate-treated necrotizing enterocolitis.

As shown in Figure 1(a), we did not observe a significant difference in body weight between the two NEC groups before the 3 days of modeling. However, during the modeling period, the body weight of the untreated NEC (NEC group) mice decreased more than that of the sodium butyrate-treated NEC (NEC+butyrate group) mice. Before sacrifice, body weight was significantly higher in the NEC+butyrate group (4.8 ± 0.2) than in the NEC group (4.3 ± 0.3, *P* = 0.012). No deaths occurred in the three groups before modeling. In the NEC group, five deaths occurred on the 1st day, and eight deaths occurred on the 2nd and 3rd days. In the NEC+butyrate group, one death occurred on the 1st day, two deaths occurred on the 2nd day, and three deaths occurred on the 3rd day. As shown in Figure 1(b), the final survival rate was significantly different among the three groups: 65% (39/60) in the NEC group, 87.5% (42/48) in the NEC+butyrate group, and 100% in the control group (*χ*^2^ = 21.28, *P* < 0.0001).

#### 3.1.2. Gross Morphology, Pathological Morphology, and Histological Scoring of Intestinal Tissue

**Figure 2:**
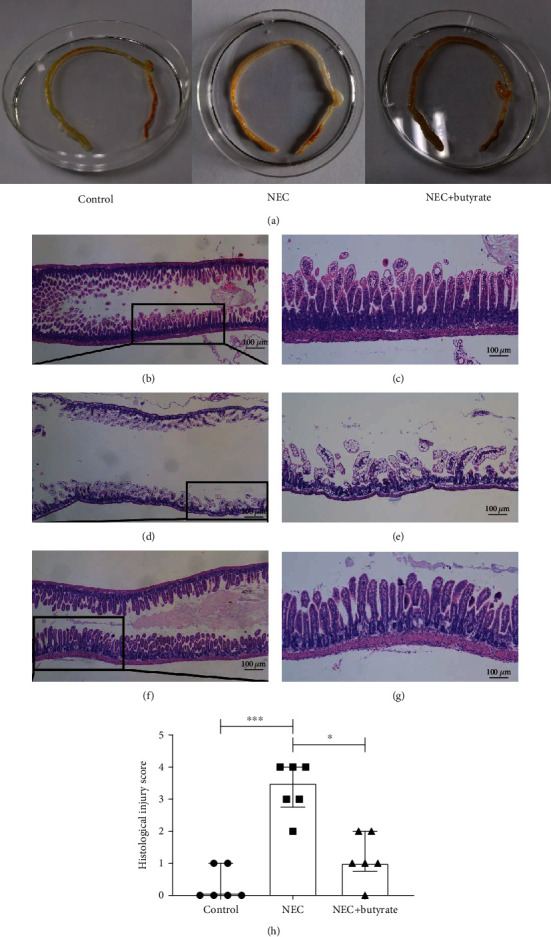
(a) Visual morphological observation of intestinal tissue from newborn mice in the three groups. (b–g) Histopathological observation of the terminal ilea in the three groups; images of HE staining observed by microscopy; (b, c) control group; (d, e) NEC group; (f, g) NEC+butyrate group. Magnification: 40x and 100x; scale bar = 100 *μ*m. (h) The intestinal histopathological injury scores in the three groups. Number of samples: control (*n* = 6), NEC (*n* = 6), and NEC+butyrate (*n* = 6). Statistical analysis: Kruskal-Wallis test. ^∗^*P* < 0.05; ^∗∗∗^*P* < 0.001.

Visual observation revealed no obvious damage in the control group but gas accumulation and droplet-like changes in the intestinal tissue in the NEC group. There was slight gas accumulation and edema but no bleeding in the NEC+butyrate group (Figure 2(a)). Upon observation under an optical microscope, the intestinal tissue structure of the control mice was clear and complete, with neatly arranged epithelial cells and a thick and continuous muscle layer without hyperemia, edema, or separation (Figures 2(b) and 2(c)). In the NEC group, the villi were degenerative and edematous; some villi were necrotic, were exfoliated, or disappeared, and the muscle layer was thin or even broken (Figures 2(d) and 2(e)). In the NEC+butyrate group, the villi were relatively intact with mild-to-moderate edema and congestion, and the muscle layer was thicker in this group than in the NEC group (Figures 2(f) and 2(g)). The median intestinal histological injury score was significantly lower in the NEC+butyrate group (1.00; IQR 0.75–2.00) than in the NEC group (3.50; IQR 2.75–4.00; *P* = 0.0458). HE-stained sections from the NEC group showed more severe damage and tissue necrosis than those from the control and NEC+butyrate groups (Figure 2(h)).

### 3.2. Sodium Butyrate Reduced the Inflammatory Reaction in Intestinal Tissue

#### 3.2.1. Changes in Inflammatory Cytokines

**Figure 3:**
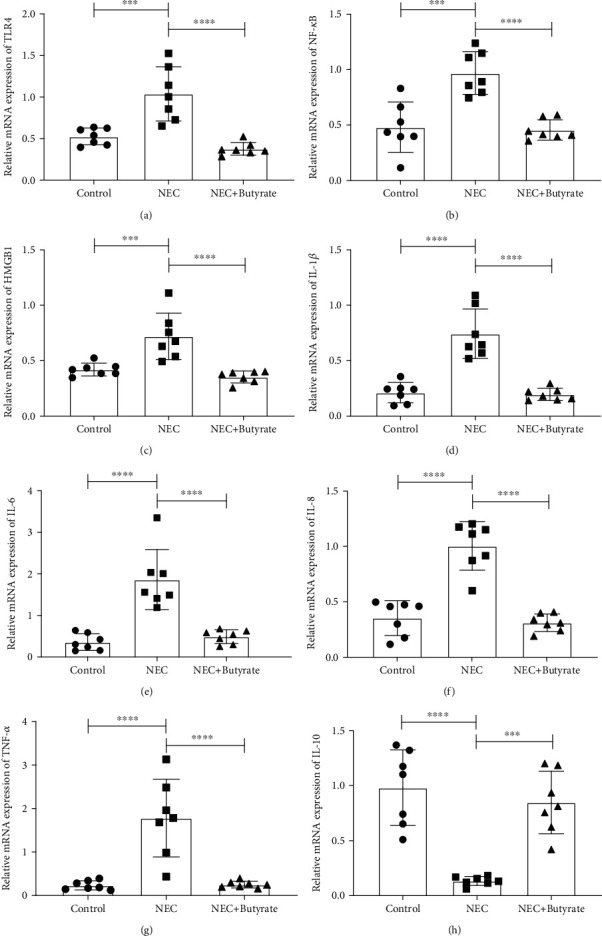
The relative mRNA expression of inflammatory cytokines in the three groups: (a) TLR4; (b) NF-*κ*B; (c) HMGB1; (d) IL-1*β*; (e) IL-6; (f) IL-8; (g) TNF-*α*; (h) IL-10. Number of samples: control (*n* = 7), NEC (*n* = 7), and NEC+butyrate (*n* = 7). Statistical analysis: one-way ANOVA. ^∗∗∗^*P* < 0.001; ^∗∗∗∗^*P* < 0.0001.

To understand the impact of sodium butyrate on inflammatory cytokines in the NEC mouse intestine, we first assessed the mRNA expression of these molecules in intestinal tissues by QPCR.  Figure 3 shows the increased secretion of the proinflammatory factors HMGB1 (Figure 3(c)), IL-1*β* (Figure 3(d)), IL-6 (Figure 3(e)), IL-8 (Figure 3(f)), and TNF-*α* (Figure 3(g)) and the markedly reduced secretion of the anti-inflammatory factor IL-10 (Figure 3(h)) in the NEC group compared with the control and NEC+butyrate groups (*P* < 0.01).

**Figure 4:**
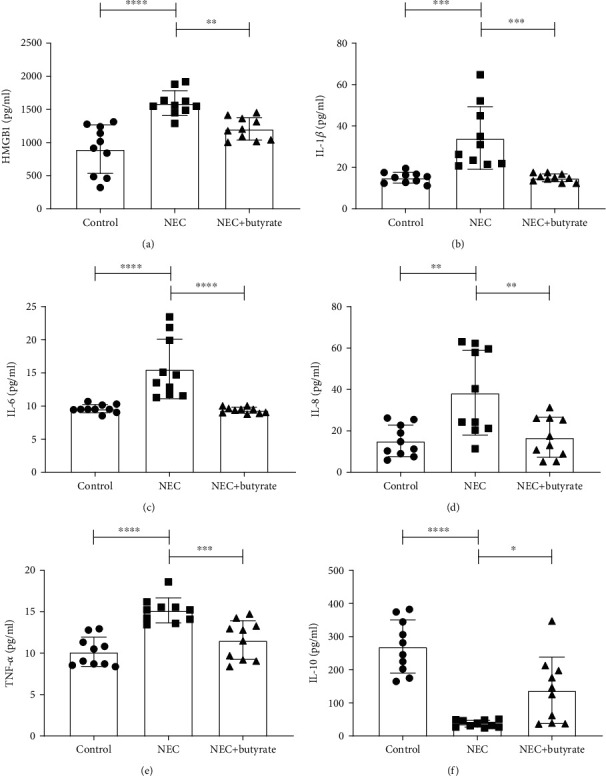
The protein concentrations of inflammatory cytokines in the three groups: (a) HMGB1; (b) IL-1*β*; (c) IL-6; (d) IL-8; (e) TNF-*α*; (f) IL-10. Number of samples: control (*n* = 10), NEC (*n* = 10), and NEC+butyrate (*n* = 10). Statistical analysis: one-way ANOVA. ^∗^*P* < 0.05; ^∗∗^*P* < 0.01; ^∗∗∗^*P* < 0.001; ^∗∗∗∗^*P* < 0.0001.

Subsequently, we assessed the protein expression of inflammatory factors by ELISA.  Figure 4 shows that the secretion of HMGB1 (Figure 4(a)), IL-1*β* (Figure 4(b)), IL-6 (Figure 4(c)), IL-8 (Figure 4(d)), and TNF-*α* (Figure 4(e)) was significantly lower in the NEC+butyrate and control groups than in the NEC group. However, the production of IL-10 (Figure 4(f)) was significantly increased (*P* < 0.05).

#### 3.2.2. Changes in TLR4 and NF-*κ*B

**Figure 5:**
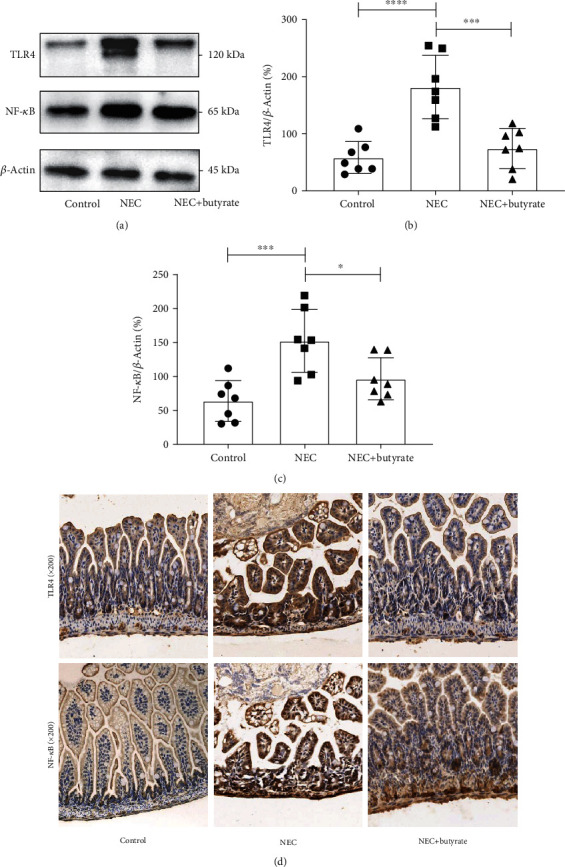
(a–c) TLR4 and NF-*κ*B protein expression levels in the three groups as determined by western blot analysis. (d) TLR4 and NF-*κ*B protein expression levels as determined by immunohistochemistry and observed under a microscope at 200x magnification. Number of samples: control (*n* = 7), NEC (*n* = 7), and NEC+butyrate (*n* = 7). Statistical analysis: one-way ANOVA. ^∗^*P* < 0.05; ^∗∗∗^*P* < 0.001; ^∗∗∗∗^*P* < 0.0001.

To determine the influence of sodium butyrate on the TLR4/NF-*κ*B intestinal signaling pathway in NEC mice, we assessed TLR4 and NF-*κ*B mRNA expression by QPCR and TLR4 and NF-*κ*B protein expression by western blotting and immunohistochemistry. Figures 3(a) and 3(b) and Figures 5(a)–5(d) show that the mRNA and protein expression levels, respectively, were increased in the NEC group. However, TLR4 and NF-*κ*B expression was decreased in the NEC+butyrate group (*P* < 0.05).

These measurement results suggest that the amplification of a series of inflammatory cascades involving HMGB1 is inhibited by butyrate treatment, thereby reducing intestinal inflammation.

### 3.3. Sodium Butyrate Partially Changed the Intestinal Flora in NEC

**Figure 6:**
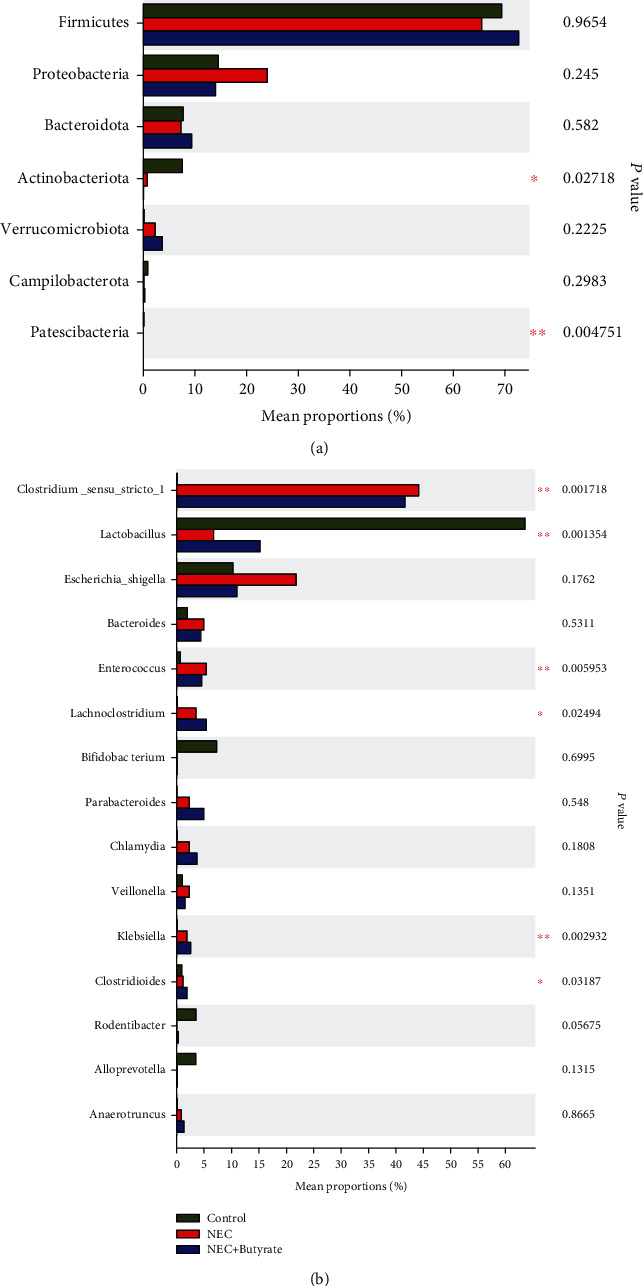
(a) Differences in the proportions of microflora constituents at the phylum level in mice in the three groups. ^∗^The top seven phyla are shown in the figure. (b) Differences in the proportions of microflora constituents at the genus level in mice in the three groups. Number of samples: control (*n* = 7), NEC (*n* = 7), and NEC+butyrate (*n* = 7). Statistical analysis: Kruskal-Wallis *H* test. ^∗^0.01 < *P* ≤ 0.05; ^∗∗^0.001 < *P* ≤ 0.01.

The composition of the microflora in each group is shown in the histogram in Figure 6. In terms of phyla (Figure 6(a)), the average relative abundance of *Firmicutes* and *Bacteroidota* was higher in the NEC+butyrate group than in the NEC group, while that of *Proteobacteria* trended toward an increase in the NEC group, but none of these differences were statistically significant (*P* > 0.05).

In terms of genera (Figure 6(b)), the relative abundance of *Clostridium_sensu_stricto_1* was obviously lower in the control group than in the NEC and NEC+butyrate groups (*P* < 0.05), and the proportion of *Clostridium_sensu_stricto_1* was lower in the NEC+butyrate group than in the NEC group. In addition, *Lactobacillus* was markedly more abundant in the control group than in the NEC and NEC+butyrate groups (*P* < 0.01); however, the abundance of *Lactobacillus* tended to increase in the NEC+butyrate group compared to the NEC group. *Escherichia*, *Shigella*, and *Veillonella* were more abundant in the NEC group than in the NEC+butyrate and control groups, although this difference was not significant. *Enterococcus* was more abundant in the NEC group than in the NEC+butyrate and control groups (*P* < 0.05).

## 4. Discussion

Studies have shown that NEC is caused by multiple factors, but the etiology is not clear. The excessive inflammatory response and dysregulation of intestinal flora are both important in NEC pathogenesis [13]. Our research shows that early intervention with sodium butyrate can help suppress inflammation and partially correct the disturbed gut flora in a NEC animal model.

Sodium butyrate, a universally acknowledged functional short-chain fatty acid compound, was previously demonstrated to have great anti-inflammatory activity through inhibiting HMGB1 in diverse diseases [14–18]. HMGB1 is an important inflammatory transmitter and proinflammatory cytokine [19], and increased HMGB1 expression is involved in the pathogenesis of NEC and can strengthen inflammation [20, 21]. The HMGB1–TLR4 axis has been shown to promote inflammation and regulate immunity in a variety of experimental models [22]. TLR4 plays a key role in inherent immunity by activating proinflammatory pathways in different cell types and inducing cytokine generation. The TLR4/NF-*κ*B signaling pathway has been considered a major modulator of the inflammatory response [23]. TLR4 signaling is required for the induction of NEC in both mice and patients [24]. Elevated levels of proinflammatory cytokines have been observed in chronic inflammatory bowel disease and correlate with the intensity of inflammation [25].

In this experiment, butyrate intervention significantly reduced the expression levels of HMGB1, TLR4, and NF-*κ*B in NEC mice. Moreover, the levels of the proinflammatory factors IL-1*β*, IL-6, IL-8, and TNF-*α* were significantly lower, and those of the anti-inflammatory factor IL-10 were higher after butyrate administration. Butyrate was found to inhibit the expression of HMGB1 [26]. Our current study revealed a decrease in proinflammatory cytokines, HMGB1, TLR4, and NF-*κ*B, and an increase in anti-inflammatory factors. We speculated that butyrate might inhibit intestinal inflammation through the HMGB1–TLR4/NF-*κ*B pathway, but further study is needed to confirm this hypothesis.

It is well known that the occurrence of NEC disrupts the bacterial flora and that decreased diversity in intestinal flora is closely related to NEC severity [27]. Our results showed that in terms of phyla, butyrate treatment of NEC mice increased the relative abundance of the beneficial bacterium *Firmicutes*, consistent with the findings of Du et al. [28]. We also found that the proportion of *Clostridium_sensu_stricto_1* slightly decreased after butyrate intervention. *Clostridium_sensu_stricto_1* is much more abundant in NEC babies than in other patients and may cause epithelial inflammation in weaned piglets [11, 29]. Furthermore, in the butyrate treatment group, the abundance of the harmful bacterium *Enterococcus* tended to decrease, while that of the beneficial bacterium *Lactobacillus* increased. The results show that butyrate administration modulates the intestinal flora composition by increasing the beneficial flora *Lactobacillus* [30] and decreasing the harmful bacteria *Enterococcus*, similar to the results reported by Li et al. [31].

## 5. Conclusions

In summary, an important finding of the current study was that sodium butyrate administration significantly reduced the expression of HMGB1 and proinflammatory cytokines and decreased intestinal inflammation. Moreover, sodium butyrate partially corrected the changes in the flora in the NEC mouse model. These results reveal that sodium butyrate has potential positive preventive effects on NEC, and the exact mechanism needs to be completely elucidated in further studies.

## Figures and Tables

**Table 1:** List of primers.

Primer	Direction	Sequence
GAPDH	Forward Reverse	TGAAGCAGGCATCTGAGGG CGAAGGTGGAAGAGTGGGAG
HMGB1	Forward Reverse	AGAGGTGGAAGACCATGTC CTCTTTCATAACGAGCCTTGTC
TLR4	Forward Reverse	TTTATTCAGAGCCGTTGGTG CAGAGGATTGTCCTCCCATT
NF-*κ*B	Forward Reverse	ATGTGCATCGGCAAGTGG CAGAAGTTGAGTTTCGGGTAG
IL-1*β*	Forward Reverse	TGGTGTGTGACGTTCCCATT CAGCACGAGGCTTTTTTGTTG
IL-6	Forward Reverse	CCAAGAGGTGAGTGCTTCCC CTGTTGTTCAGACTCTCTCCCT
IL-8	Forward Reverse	CAAGGCTGGTCCATGCTCC TGCTATCACTTCCTTTCTGTTGC
IL-10	Forward Reverse	GCCGTCATTTTCTGCCTCAT GCTTCCCTATGGCCCTCATT
TNF-*α*	Forward Reverse	CCAAAGGGATGAGAAGTTCC CTCCACTTGGTGGTTTGCTA

## Data Availability

The datasets generated for this study are accessible from the corresponding author upon request.
